# Speech Identification with Temporal and Spectral Modification in Subjects with Auditory Neuropathy

**DOI:** 10.5402/2012/671247

**Published:** 2012-09-09

**Authors:** Vijaya Kumar Name, C. S. Vanaja

**Affiliations:** ^1^Department of Audiology, All India Institute of Speech and Hearing, Manasagangothri, Mysore, Karnataka, 570006, India; ^2^Department of Audiology & Speech Language Pathology, Bharati Vidyapeeth University School of Audiology and Speech Language Pathology, Dhankawadi, Pune, Maharashtra 410021, India

## Abstract

*Background*. The aim of this study was to investigate the individual effects of envelope enhancement and high-pass filtering (500 Hz) on word identification scores in quiet for individuals with Auditory Neuropathy. *Method*. Twelve individuals with Auditory Neuropathy (six males and six females) with ages ranging from 12 to 40 years participated in the study. Word identification was assessed using bi-syllabic words in each of three speech processing conditions: unprocessed, envelope-enhanced, and high-pass filtered. All signal processing was carried out using MATLAB-7. *Results*. Word identification scores showed a mean improvement of 18% with envelope enhanced versus unprocessed speech. No significant improvement was observed with high-pass filtered versus unprocessed speech. *Conclusion*. These results suggest that the compression/expansion signal processing strategy enhances speech identification scores—at least for mild and moderately impaired individuals with AN. In contrast, simple high-pass filtering (i.e., eliminating the low-frequency content of the signal) does not improve speech perception in quiet for individuals with Auditory Neuropathy.

## 1. Introduction

Conventional hearing aids amplify acoustic signals to make sounds audible to hearing-impaired individuals. Their basic structure consists of a microphone, an amplifier, a receiver (speaker), and a power supply. The amplifier is the major component that amplifies the input signal. Two types of amplification schemes are typically used in hearing aid design. The first scheme is the linear amplification, in which a set amount of gain is applied to the input signal. In this design, the maximum output is limited by peak clipping, which causes various forms of distortion and reduces the intelligibility and subjective quality of speech. The second scheme is a nonlinear amplification, which reduces gain as the output or input approach maximum values. In this scheme, amplitude compression is implemented by an analog circuit or by a digital processing algorithm to reduce the gain of the instrument when either the input or output exceeds a predetermined level [1]. This type of amplification results in a wider dynamic range, making soft sounds audible without making loud sounds uncomfortably loud [2]. However, amplitude compression also changes the temporal properties of the original speech signal, which may reduce speech intelligibility.

Conventional hearing aids are not effective for all hearing impairments [3, 4]. The primary function of a conventional hearing aid is to amplify and make the speech signal audible within the hearing threshold and loudness tolerance levels of an individual with hearing loss. These systems are typically effective for hearing impairments caused by a loss of amplification function in the ear, such as with cochlear hearing loss due to outer hair cell loss and/or damage. However, even sophisticated hearing aids are limited in their ability to address other, less common types of hearing loss, such as hearing loss caused by neural fiber removal (via tumor operations), which leave patients with little or no residual hearing, or losses caused by damage to the inner hair cells, neural pathways, or brainstem, which affects supra-threshold processing and introduces sound distortion. 

Auditory Neuropathy (AN) can involve loss of inner hair cells (IHC), dysfunction of the IHC-nerve synapses, neural demyelination, axonal loss, or possible combinations of the above. Clinically, these pathologies may be mixed with traditional cochlear impairment involving OHC's and/or central processing disorders involving the brainstem and cortex. Because one possible neural mechanism underlying AN is desynchronized discharge of the auditory nerve fibers, AN has also been termed “Auditory Dys-synchrony” [5]. AN can cause sound attenuation, but, more significantly, it causes sound distortion, which cannot be compensated by conventional hearing aids [3, 4]. Therefore, new signal processing strategies should be developed to specifically address the issue of sound distortion for these participants. 

Clinical and psychoacoustic testing on individuals with AN has been conducted to investigate the root causes of sound distortion [6]. Pure-tone audiograms of AN subjects show a global trend opposite to regular hearing impairment—high thresholds at low frequencies but low or relative normal thresholds at high frequencies—implying that amplifying energy at high frequencies or transposing high-frequency components to the low-frequency range may not help. Test results from the temporal modulation transformation function (TMTF) show that individuals with AN have poorer temporal modulation discrimination ability than normal-hearing and other hearing-impaired people. This again implies that conventional hearing aids will not be fully effective for these individuals since their degraded temporal modulation is not being compensated. In addition, data from gap detection tests shows lower gap discrimination ability in individuals with AN than other hearing impairments, which also supports the notion that AN patients have impaired temporal processing abilities. Therefore, it appears that new strategies might be developed based on these clinical and psychoacoustic data to solve the problem of sound distortion in AN. 

One strategy directly stemming from the TMTF is to increase the modulation index in each frequency band to compensate for the temporal modulation loss due to desynchronized discharges in the auditory nerve fibers of individuals with AN. This can be implemented over each extracted envelope in each frequency band by directly increasing the amplitude of peaks and decreasing the amplitude of troughs in a local temporal range. However, Fu and Shannon [7] pointed out that enhancement of the envelope using such a “power law expansion scheme” tends to modify consonant-to-vowel ratio when the overall RMS of the expanded and control (unexpanded) stimuli are equated and leading to sound distortion in the expanded sound. Apoux et al. [8] applied an envelope enhancement scheme, which enhances the consonantal portion of the signal while attenuating the vowel portion. They observed a significant improvement in identification scores when the envelope-enhanced stimuli were presented to individuals with cochlear hearing loss in the presence of background noise. Research has indicated that “clear speech” also improves speech perception in individuals with cochlear hearing loss. One of factors linked to increased intelligibility of clear speech in cochlear hearing loss is increased consonant-vowel ratio [9–11]. 

Zeng and Liu [12] reported that subjects with AN showed improved performance in quiet as well as in noise when clear speech was presented. This improvement was attributed to the enhanced temporal envelopes found in clear speech. Starr et al. [13] have shown that (artificially) enhancing the temporal envelope improves consonant identification in quiet for individuals with AN. From the simulation studies it is understood that the reduced ability to follow amplitude variations leads to the loss of distinction between the vowel and consonant causing significant impairment in speech perception [6, 14]. If this is true, then envelope enhancement should enhance the salient cues for speech perception in individuals with AN, across both quiet and noisy environments. This study was undertaken to investigate this hypothesis in quiet environments. 

In addition to the previously identified temporal resolution deficits that typify AN, psychoacoustical studies have demonstrated that individuals with AN have impaired frequency discrimination. It has been reported [6, 15] that frequency discrimination is poorer for low frequency sounds and performance improves as the frequency is increased, reaching near normal values at 4000 Hz. Consistent with these findings, Rance et al. [16] and Starr et al. [13] observed that individuals with AN demonstrate better identification for phonemes that lie in relatively high-frequency ranges than for those phonemes that lie in relatively low-frequency ranges. Zeng et al. [17] have hypothesized that eliminating the low-frequency content of speech signal or shifting the low frequency content to high frequencies may improve speech perception and reduce undesirable masking in individuals with AN.

## 2. Method

### 2.1. Participants

Twelve individuals (six males and six females) who have been diagnosed with AN participated in the study.  Table 1 shows the audiological profile of the participants. The age of the participants ranged from 12 to 40 years with a mean of 25 years. All the participants were native speakers of Kannada, a Dravidian language spoken in a southern state of India. The pure-tone average (average of pure tone thresholds at 500, 1000, and 2000 Hz, 4000 Hz, and 8000 Hz) of the participants ranged from 15 to 55 dB HL. A majority of the participants had symmetrical hearing loss with rising audiometric configuration. The middle ear acoustic reflexes (both ipsilateral and contralateral) and the auditory brainstem responses were absent in all the participants. None of the participants showed improvement in speech recognition scores with a conventional linear gain behind ear hearing aid.

#### 2.1.1. Ethical Considerations

In the present study, all the testing procedures were approved by institutional review board. The procedures involved in the present study were noninvasive and all the procedures were explained to the patients and their family members before testing, and informed consent has been taken from all the patients and their family members for participating in the study.

### 2.2. Stimuli

The speech stimuli used in the present study were taken from bi-syllabic wordlists in Kannada, developed by Yathiraj and Vijaylakshami [18]. This test contains four word lists, each with 25 bi-syllabic words, which are phonetically balanced and are equally difficult. The words were spoken in conversational style by a female native speaker of Kannada. They were digitally recorded in an acoustically treated room, on a data acquisition system using 44.1 kHz sampling frequency and 32-bit analog to digital converter. 

### 2.3. Signal Processing

**(i) Envelope Enhancement**

The enhancement procedure used in the present study was similar to that used by Apoux et al. [8] for individuals with cochlear hearing loss. The original speech stimuli, *X*(*t*), was expanded/compressed using MATLAB-7.

The signal *X*(*t*) was divided into 4 bands using band pass filters (third order Butterworth) with cut-off frequencies of 150–550, 550–1550, 1550–3550, and 3550–8000 Hz. The temporal envelopes *E*(*t*) were extracted from each band by full-wave rectification and low pass filtering (third-order Butterworth) with a cut-off frequency 32 Hz. This was chosen based on the result of an earlier investigation, where it produced the best outcome among several different cut-off frequencies used [13]. The temporal envelope *E*(*t*) was raised to the power of *f*, with *f* ranging from a highly expansive value (*f*_max⁡_ = 4) to a highly compressive value (*f*_min⁡_ = 0.3) as a function of the instantaneous envelope amplitude, *Ei*. The exponent *f* was computed via a decreasing, exponential function of instantaneous envelope amplitude value *Ei*, and was set such that: (i) maximum enhancement (*f*_max⁡_ = 4) was applied to the lowest envelope amplitude *E*_min⁡_ and (ii) maximum compression (*f*_min⁡_ = 0.3) was applied to the highest envelope amplitude value. The formula used for computing the *f is *given as follow:

(1) $$\begin{array}{c} f_{i}=e^{-(F_{i}-F_{\min})/\tau }\tau \left(f_{\max}-f_{\min}\right)+f_{\min}. \end{array}$$

**Figure 1:**
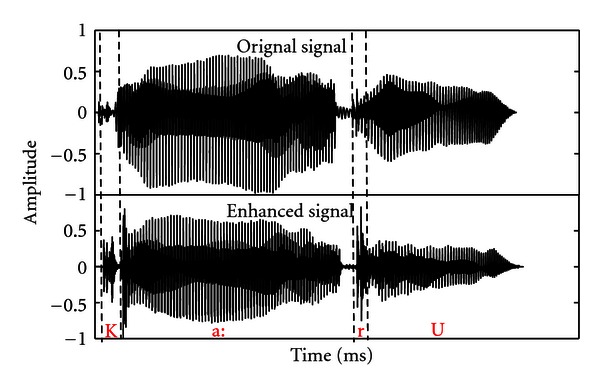
Waveform of the original signal (upper panel) and enhanced signal (lower panel) for word/ka: r u/. The dotted line indicates the vowel portion of the signal, and striated line indicates the consonantal portion of the signal.

In this equation, *τ* was a constant, (0.5 for each word) and *E*_min⁡_, the minimum envelope amplitude value, was computed over the whole signal duration. A correction factor was then obtained for all the bands by computing the ratio of expanded and original envelope for each sample. The correction factor obtained was then multiplied with the original band pass signal at each corresponding point in time, and finally the resulting bands were added at output and low pass filtered (3rd order Butterworth) with a cutoff frequency of 8000 Hz. The RMS amplitude of the expanded signals was then equated to that of the unprocessed signals. Three word lists were processed for envelope enhancement.  Figure 1 shows the waveform of signal with and without envelope enhancement.

**(ii) High-Pass Filtering**

Bisyllabic words were high-pass filtered (fourth order Butterworth) with a cutoff frequency of 500 Hz. The filtering was done using the MATLAB-7 program. The RMS amplitude of the filtered signals was then equated to that of the unprocessed signals. All four word lists were passed through the filters.

### 2.4. Procedure

The participants listened to speech tokens individually in a double-walled, acoustically treated room where the ambient levels were within permissible limits [19]. The speech stimuli were played manually from a PC at 44.1 kHz sampling rate and routed to a calibrated [20] diagnostic audiometer (Madsen OB-922 with speaker). The participants listed to the signal from the loudspeaker of the audiometer at a distance of one meter with 0° azimuth. The presentation level of the stimulus was 40 dB SL (re: Speech Recognition Threshold). Each participant listened to a total of four lists: one unprocessed, two filtered, and one envelope enhanced. The order of presentation of the lists was randomized across the participants. Participants had to repeat the speech tokens heard by them. The speech recognition scores were calculated by counting the number of words correctly repeated.

### 2.5. Data Analysis

 Statistical analyses of the data were performed using SPSS-16. Paired sample “*t*” testing was carried out to assess whether there was a significant difference between the scores obtained in the unprocessed, filtered, and envelope-enhanced conditions. Pearson's product moment correlation coefficient was calculated to investigate if there was a correlation between pure-tone threshold and word identification scores in the three conditions. Because a majority of the participants had symmetrical hearing loss, only right ear thresholds were used for this analysis.

## 3. Results

### 3.1. Word Identification with Envelope Enhanced Speech

**Figure 2:**
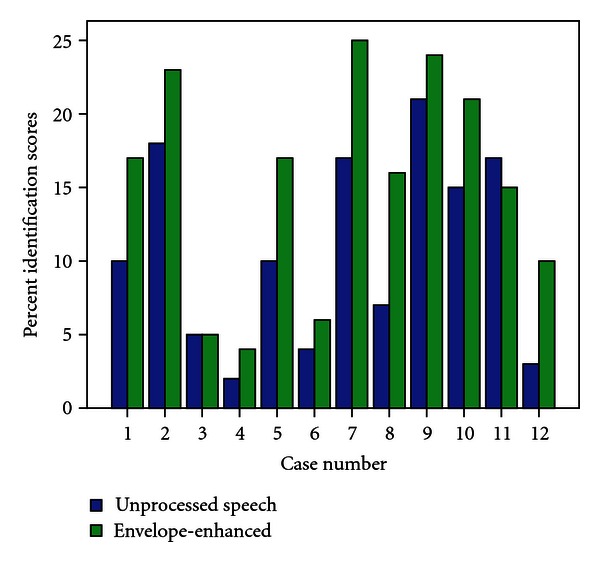
Word identification scores for unprocessed (open bar) and envelope-enhanced (filled) speech.

Figure 2 presents the identification scores for unprocessed and envelope-enhanced words for the AN test subjects. The mean identification score for envelope-enhanced speech was 61.3% with a standard deviation (SD) of 33.2%, whereas the mean identification score for unprocessed speech was 43.6% with an SD of 27%. The improvement observed for envelope-enhanced speech ranged from 8% to 36% with a mean improvement of 18.3%. To assess effect of gender on identification scores in unprocessed and envelope-enhanced condition, a paired sample “*t*” test was performed and results showed no significant effect of gender on identification for unprocessed (*t* = 0.44, *P* = 0.66) and envelope-enhanced (*t* = −0.31, *P* = 0.75). As there was a significant effect of gender the data from both males and females is combined for further analysis.

 The paired sample “*t*” test indicates that the improvement observed for envelope enhanced speech was statistically significant (*t* = −4.38, *P* < 0.01). Pearson's correlation coefficient revealed that there was no significant correlation between word identification scores for the unprocessed signal and pure-tone average threshold (*r* = −0.37, *P* = 0.6). Scores for envelope enhanced speech also did not show a significant correlation with pure-tone average threshold (*r* = −0.13, *P* = 0.23). 

### 3.2. Word Identification with Filtered Speech

**Figure 3:**
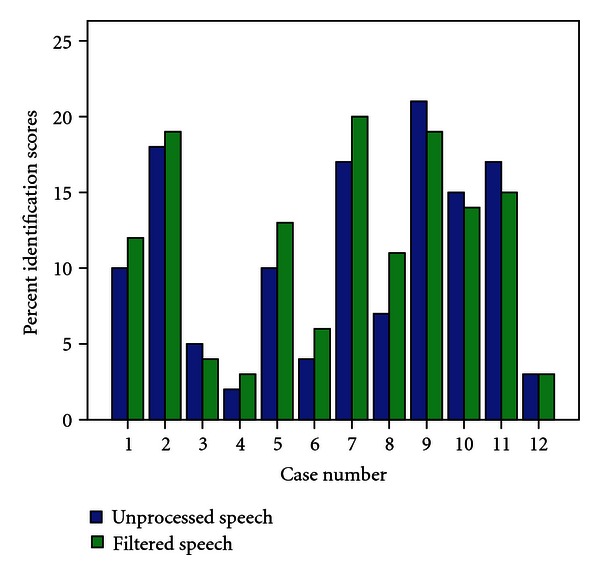
Word identification scores for unprocessed (open bar) and filtered speech (filled bar).

 Percent identification scores for all participants are presented in Figure 3. It can be noted from the figure that five participants showed a small improvement in identification with filtered speech, while seven participants showed no improvement or deterioration in performance with filtered speech. The mean identification score for high-pass filtered speech at 500 Hz was 45.6% with standard deviation of 25.4%, whereas mean identification score for unprocessed speech was 43% with standard deviation of 26.3%. To assess effect of gender on identification scores in unprocessed and envelope-enhanced condition, a paired sample “*t*” test was performed and results showed no significant effect of gender on identification for filtered speech (*t* = 0.29, *P* = 0.79). As there was significant effect of gender, the data from both males and females is combined for further analysis. 

Paired sample “*t*” testing revealed that the mean difference in scores between unprocessed speech and high pass filtered speech was not statistically significant (*t* = 1.54, *P* = 0.85). Pearson's correlation coefficient revealed that there was no significant correlation between word identification scores for unprocessed speech and pure-tone average threshold (*r* = −0.37, *P* = 0.6). Scores for envelope enhanced speech also did not show a significant correlation with pure-tone average threshold (*r* = −0.13, *P* = 0.23). 

## 4. Discussion

### 4.1. Word Identification with Envelope-Enhanced Speech

One of the primary goals of the present study was to compare the word identification scores for unprocessed and envelope-enhanced speech in individuals with AN. The results of the present study reveal that envelope enhanced speech improved word identification in all but four participants with AN. Starr et al. [13] observed similar results for consonant identification in a earlier study. Zeng and Liu [12] have observed similar results for clear speech with AN. 

 Speech understanding difficulties in individuals with AN are mainly attributed to an impairment in processing the amplitude modulations of the speech signal [6, 15]. Impaired processing of amplitude variations blurs the distinction between consonant and following vowel, which limits the perception of salient consonantal cues [14]. Dubno and Levitt [21] observed that in both quiet and noise, normal-hearing subjects placed more importance on the consonantal energy and consonant-to-vowel ratio, respectively, in identifying the consonants. Studies in normal hearing and those with cochlear hearing loss have shown greater improvement in speech identification in the presence of noise when the processing strategy enhanced the consonantal portion of the signal while attenuating the vowel portion [8]. These observations suggest that when temporal processing is impaired, so too is the ability to identify consonants. By enhancing the amplitude modulations of the speech signal by using the compression/expansion scheme employed in the present study, the consonantal portion of speech signal was also enhanced, leading to an improvement in word identification in individuals with AN.

In the present study four participants did not improve with envelope enhancement. However, these individuals had very poor unprocessed speech identification scores. Zeng et al. [6] and Narne and Vanaja. [15] have demonstrated high levels of correlation between speech identification scores and amplitude modulation detection capabilities. They also reported that some individuals with AN even have difficulty in perceiving amplitude fluctuations as high as 100%. Zeng et al. [6] categorized individuals with AN into different groups based on the TMTF threshold. These groups were mild, moderate, severe, and profound. TMTF thresholds were not assessed in the present study, but it is likely that the degree of TMTF impairment was greater in individuals who showed no or minimal improvement in this study, and that the envelope enhancement was insufficient to overcome this degree of impairment.

**Figure 4:**
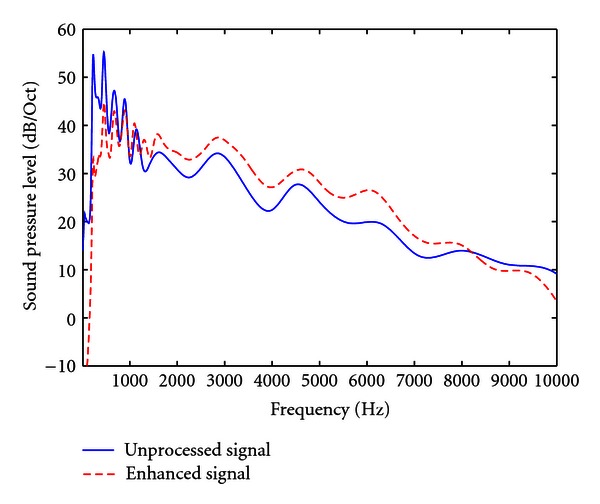
Long-term spectrum of enhanced (black) and original (gray) signals for all the stimuli presented.

Figure 4 depicts the long-term average speech spectrum of unprocessed and envelope-enhanced stimuli, normalized for long-term RMS level. The long-term spectrum was computed using a 50 ms nonoverlapping Hamming window. The envelope enhanced speech shows 3–5 dB higher energy in the mid-frequency region compared to the unprocessed speech. However, the improved word identification for envelope enhanced speech cannot be attributed to the small increase in intensity in the mid-frequency region, as the correlation analysis between pure-tone thresholds and word identification scores and also the results of hearing aid assessment indicate that audibility was not a factor affecting word identification scores in these individuals. Earlier studies have also reported that audibility is not a factor affecting speech identification scores in a majority of the individuals with AN [21]. In addition, the long-term spectra of clear speech show more energy concentration in the mid-frequency region [11]. However, Zeng and Liu [12] attribute the advantage observed for clear speech in individuals with AN to the enhanced envelopes of clear speech. Thus, based on the results of the present study and earlier reports, it can be concluded that envelope enhancement improved word identification in individuals with AN.

### 4.2. Word Identification with Filtered Speech

The word identification for filtered speech was not significantly different from unprocessed speech. It has been reported in the literature that high pass filtering a speech signal at 500 Hz does not have major effects on identification scores in normal hearing and those with cochlear hearing loss [22]. Results of the present study clearly demonstrate that simply eliminating the low-frequency information by high pass filtering of the speech signal did not improve the speech perception in individuals with AN in quiet. Furthermore, filtering of the speech signal caused deterioration in speech identification scores in some individuals with AN. A close look at the data suggests that even individuals with low thresholds at low frequency did not benefit from filtering of the speech signal. An independent sample “*t*” test showed there is no significant difference (*t* = 0.39, *P* = 0.73) in identification scores for filtered speech between low-frequency hearing loss participants and those with other audiogram patterns. This clearly suggests that simply eliminating the low frequency elements of a signal or emphasizing high frequencies does not compensate for poor speech perception in AN, at least in quiet. This suggests that the cause of their speech understanding difficulties is mainly due to temporal impairment. Further, low frequency hearing loss and poor processing of the signal in low-frequencies is also due to temporal impairment [23]. However, the importance of eliminating low frequency information in the presence of noise still needs to be investigated. 

## 5. Conclusion

 Enhancing the speech signal with the compression/expansion scheme, which enhances the consonant portion of the signal, significantly improved speech identification scores in quiet for individuals with AN. In contrast, high pass filtering does not improve the speech identification scores in individuals with AN in quiet. These results suggest that the compression/expansion signal processing strategy enhances speech identification scores—at least for mild and moderately impaired individuals with AN.

## Figures and Tables

**Table 1:** Audiological profile of individuals with auditory neuropathy.

Subject no.	Age/sex	Pure-tone average in dB HL (Right ear)	Pure-tone average in dB HL (Left ear)	Speech identification scores in sound filed	Peripheral neuropathy
AN1	12 ys/M	22.50	25.5	40	No
AN2	20 ys/F	28.75	30.5	72	No
AN3	15 ys/F	26.25	25.5	20	Not done
AN4	39 ys/F	31.25	32.5	8	No
AN5	12 ys/M	40.00	42.5	40	No
AN6	24 ys/M	32.50	35.5	16	Present
AN7	27 yr/F	26.25	29.5	68	No
AN8	20 yr/M	47.50	46	28	No
AN9	18 yrs/M	8.75	10	84	No
AN10	20 yrs/M	40.00	35.5	48	Not done
AN11	20 yrs/F	27.50	22.5	12	No
AN12	19 yrs/F	21.25	23.5	68	No
